# Centre Versus Surgeon Volume in Pancreatic Surgery

**DOI:** 10.3390/jpm16100502

**Published:** 2026-09-28

**Authors:** Mario Annecchiarico, Giuseppe Loiaco, Claudia Cirillo, Antonio Antonino, Angela Romano, Antonio Varricchio, Francesco Carafa, Pellegrino Gambardella, Giovanni Meola, Giulio Argenio

**Affiliations:** 1 General and Oncological Surgery Unit, Department of Oncological Sciences, San Pio Hospital, Via Pacevecchia 57, 82100 Benevento, Italy; mario.annecchiarico@aornsanpio.it (M.A.); claudia.cirillo@aornsanpio.it (C.C.); antonio.antonino@aornsanpio.it (A.A.); angela.romano@aornsanpio.it (A.R.); antonio.varricchio@aornsanpio.it (A.V.); francesco.carafa@aornsanpio.it (F.C.); p.gambardella@student.unisi.it (P.G.); g.meola2@studenti.unimol.it (G.M.); giulio.argenio@aornsanpio.it (G.A.); 2 Clinical and Translational Oncology PhD Programme, Scuola Meridionale Superiore, 80138 Naples, Italy; 3 Department of Medical and Surgical Sciences and Neurosciences, University of Siena, 53100 Siena, Italy

**Keywords:** pancreatic surgery, pancreatoduodenectomy, distal pancreatectomy, total pancreatectomy, centre volume, surgeon volume, robotic surgery, failure to rescue, centralisation

## Abstract

**Background/Objectives**: Pancreatic resections have different technical demands and rescue requirements, yet centre and surgeon volume are often treated as interchangeable exposures. Their relative contributions are particularly relevant as robotic pancreatic surgery expands. **Methods**: PubMed/MEDLINE was searched from inception to 24 August 2026 for systematic reviews, population-based and multilevel studies, failure-to-rescue analyses, consensus statements, learning-curve studies, and randomised or multicentre evidence concerning pancreatoduodenectomy, distal pancreatectomy, total pancreatectomy, and robotic pancreatic surgery. **Results**: Surgeon experience was consistently related to technical execution after pancreatoduodenectomy, whereas centre volume showed an additional association with 90-day mortality, readmission, and failure to rescue. Contemporary interaction analyses indicate that a high-volume surgeon does not reliably compensate for a low-volume hospital. Associations with centre volume were strongest for pancreatoduodenectomy and total pancreatectomy; evidence for standard distal pancreatectomy was less consistent, although high-volume centres achieved lower failure-to-rescue rates. Robotic pancreatoduodenectomy introduced concurrent surgeon, team, and centre learning curves. More favourable outcomes during robotic adoption were reported in established open pancreatic programmes using structured training, proctoring, case selection, and prospective audit. Randomised robotic evidence was generated in credentialled high-volume centres and should not be extrapolated uncritically to early programmes. **Conclusions**: Pancreatic volume policy should be operation-specific. Surgeon experience is more closely associated with technical performance, whereas centre-level capability is more strongly associated with complication recognition and rescue. Referral and robotic credentialing should therefore integrate procedure-specific experience, audited outcomes, rescue capability, learning-curve status, and access, rather than rely on a single annual threshold.

## 1. Introduction

Pancreatic surgery is not a single exposure. Pancreatoduodenectomy (PD), distal pancreatectomy (DP), and total pancreatectomy (TP) differ in reconstruction, haemorrhagic and fistula-related risk, metabolic consequences, postoperative surveillance, and rescue requirements. Pooling them under pancreatic resection may therefore obscure procedure-specific volume–outcome relationships. The inverse association between hospital volume and operative mortality is among the most reproducible observations in surgical health-services research [1,2,3,4,5,6], while surgeon volume is also associated with mortality and morbidity after PD [7,8]. These exposures are correlated but not interchangeable. Individual experience influences technical execution, whereas the wider pancreas programme supports diagnosis, interventional radiology, therapeutic endoscopy, critical care, reoperation, and longitudinal oncological care.

The distinction has immediate implications for centralisation. If surgeon volume fully explained outcomes, an experienced pancreatic surgeon might reproduce results in any adequately equipped hospital. If centre capability retains an independent association, concentrating only on the operator is insufficient. Recent analyses that model both levels support the latter interpretation, especially for 90-day mortality and readmission after PD for pancreatic ductal adenocarcinoma (PDAC) [9,10]. Robotic pancreatic surgery intensifies this distinction. The platform may facilitate suturing and dissection, but it does not remove fistula, haemorrhage, or rescue risk, and introduces concurrent learning curves for the surgeon, team, and institution. This review examines centre and surgeon volume specifically within pancreatic surgery, separates the evidence by operation, and evaluates how robotic adoption should modify referral and credentialing.

## 2. Literature Search and Evidence Synthesis

A structured narrative search of PubMed/MEDLINE was conducted from database inception to 24 August 2026. Search concepts combined pancreatectomy, pancreatic resection, pancreatoduodenectomy, distal pancreatectomy, or total pancreatectomy with hospital, centre, surgeon, operator, provider, or team volume; centralisation, regionalisation, failure to rescue, transfer, textbook outcome, access, or waiting time; and minimally invasive, laparoscopic, robotic, learning curve, training, proctoring, or credentialing. Reference lists of eligible reviews, international guidelines, and key comparative studies were screened, followed by forward citation searching. The complete PubMed/MEDLINE search strategy is provided in the Supplementary Materials.

Priority was assigned to systematic reviews and meta-analyses, national or regional registries with risk adjustment, analyses that modelled surgeon and hospital volume concurrently, pancreas-specific failure-to-rescue studies; operation-specific evidence for PD, DP, and TP; and randomised, nationwide, or international multicentre studies of minimally invasive and robotic pancreatectomy. When multiple reports addressed the same question or included overlapping populations, preference was given to the most contemporary, procedure-specific, and methodologically informative analysis, particularly studies incorporating risk adjustment or concurrent surgeon–centre modelling. Earlier or overlapping studies were retained when they provided distinct outcomes, methodological perspectives, or historical context. Studies reporting mixed hepatopancreatobiliary outcomes without separable pancreatic data were not used as primary evidence.

Throughout this review, centre volume was used as the overarching term. Hospital volume is retained when it reflects the exposure defined in the original study, whereas programme volume refers primarily to procedure-specific minimally invasive or robotic activity.

No de novo meta-analysis was undertaken because volume definitions, procedures, risk adjustment, and outcome windows were heterogeneous. Given the narrative design, no formal study-level risk-of-bias instrument was applied. The principal volume–outcome evidence was predominantly observational and therefore remained susceptible to residual confounding, referral and case-selection bias, exposure misclassification, variation in coding and outcome ascertainment, and collinearity between surgeon and centre volume. Greater interpretive weight was therefore given to risk-adjusted studies, analyses modelling surgeon and centre volume concurrently, and prospective or randomised evidence when available. The review was not prospectively registered and should be interpreted as a critical narrative synthesis.

## 3. Why Volume Must Be Interpreted Within Pancreatic Surgery

### 3.1. Exposure Is Procedure-Specific

Annual pancreatectomy volume, annual PD volume, cumulative career experience, recent case frequency, and robotic platform experience are distinct constructs. Frequent DP does not confer equivalent exposure to pancreaticojejunostomy, uncinate dissection, or portal–mesenteric venous resection, and a centre performing many standard DPs may not reproduce the rescue pathway required after PD or TP. Accordingly, high-volume definitions range from fewer than 10 to more than 60 pancreatic resections or PDs per year, while surgeon thresholds commonly range from approximately 4 to 12 PDs annually [2,3,4,5,6,8]. An analysis of 61,920 pancreatectomies identified an optimised threshold of 21 procedures per year, but mortality continued to fall above it [6], arguing against a single biological cut-off. Cumulative experience may matter independently of annual frequency. In 1003 PDs at a high-volume institution, at least 50 previous procedures were associated with lower morbidity, fistula, blood loss, and operating time, while experienced surgeons had comparable outcomes despite different annual volumes [11]. National credentialing data further showed that related hepatopancreatobiliary experience partly compensated for moderate, but not extremely low, PD volume [12]. Volume is therefore a surrogate for exposure and programme maturity rather than proof of competence.

### 3.2. Outcomes Identify Different Mechanisms

The outcome chosen determines which provider level is being tested. Blood loss, conversion, operating time, anastomotic quality, and selected complications are proximal to technical performance, whereas mortality, readmission, failure to rescue (FTR), and receipt of adjuvant therapy reflect the combined operative and organisational pathway. Textbook outcome integrates several dimensions, and long-term survival is further influenced by tumour biology, neoadjuvant selection, pathology, and systemic therapy. Although the separation is not absolute, surgeon and centre volume should be modelled together and matched to outcomes with a plausible causal pathway. Omitting either level risks misattributing its effect. This framework is summarised in Figure 1.

## 4. Centre and Surgeon Volume by Pancreatic Procedure

### 4.1. Pancreatoduodenectomy

PD dominates the evidence base because it combines complex dissection, reconstruction, high morbidity, and a substantial need for rescue. Foundational Medicare analyses reported operative mortality of 16.3% in very-low-volume versus 3.8% in very-high-volume hospitals after pancreatic resection [1]. In the subsequent surgeon analysis, low surgeon volume was associated with 3.61-fold higher adjusted odds of death, and surgeon volume statistically explained 55% of the hospital-volume association [7]. These older administrative data established that both levels matter but could not characterise modern pancreatic pathways. Later meta-analyses remained directionally consistent. The surgeon-volume meta-analysis of 36,449 PDs reported mortality of 2.4% with high-volume surgeons versus 6.7% with low-volume surgeons, together with lower morbidity and shorter stays [8]. A broader 2023 review estimated lower mortality for both high-volume hospitals and high-volume surgeons, while emphasising heterogeneity in thresholds and adjustments [5]. These associations remain observational and partly reflect referral, selection, and coding.

Studies that model both levels are more informative. Joint analyses generally found the best outcomes when a high-volume surgeon worked in a high-volume hospital [13,14,15]. In a single high-volume centre, low surgeon volume increased fistula rates but not mortality or major morbidity, a pattern compatible with institutional rescue after a technical complication [16]. In SEER–Medicare data, surgeon volume was not associated with major complications, 90-day mortality, or readmission after matching within hospital-volume strata, whereas hospital volume remained the stronger predictor [10]. In the most contemporary direct comparison of 50,519 PDs for PDAC, outcomes were best when both surgeon and hospital volume were high, whereas a high-volume surgeon did not fully offset the mortality associated with a low-volume hospital. Low surgeon volume within a high-volume hospital was not independently associated with mortality [9]. This does not render the operator irrelevant; rather, these joint analyses suggest that centre-level factors may be more strongly associated with short-term mortality than surgeon volume alone.

The centre–mortality curve is not indefinitely linear. English national data showed no mortality difference between centres performing 36–60 and more than 60 PDs per year after mortality fell markedly over time [17], whereas Dutch data showed progressively lower 90-day mortality and more complete nodal assessment at higher volumes [18]. Together, these findings support a graded association with diminishing marginal returns rather than a universal threshold. The principal studies directly evaluating or jointly modelling centre and surgeon volume are summarised in Table 1.

### 4.2. Distal Pancreatectomy

DP should not inherit PD-based policy without qualification. It usually avoids pancreaticoenteric reconstruction and has lower mortality, although clinically relevant POPF, haemorrhage, infected collections, and reoperation remain important. A French nationwide study of 3314 DPs found neither a centre-volume cut-off nor a significant mortality difference across volume groups [19], whereas an analysis of 10,632 DPs associated at least 20 annual pancreatectomies with lower adjusted mortality and FTR [20]. The findings are not necessarily contradictory because low event rates limit mortality analyses, procedure-specific and aggregate pancreatic volume are not equivalent, and FTR may discriminate centre performance more sensitively. Registry data likewise showed superior PD outcomes at pancreas-targeted hospitals without a comparable advantage for DP [21]. These data suggest that volume sensitivity within DP increases with procedural complexity.

### 4.3. Total Pancreatectomy

TP combines the operative risks of major pancreatic resection with permanent endocrine and exocrine insufficiency. It therefore tests both perioperative rescue and longitudinal programme capability. In a prospective pan-European study of 277 elective TPs across 43 centres, in-hospital mortality was 4% in centres performing at least 60 PDs annually versus 10% below that level. Centre PD volume below 60 was independently associated with mortality [22]. Although the threshold was exploratory and based on only 13 in-hospital deaths, it supports the concentration of TP in centres with mature PD, diabetes, nutrition, and readmission pathways.

### 4.4. Oncological Pathway Outcomes

For PDAC, the value of a pancreas centre extends beyond the operation. Centralisation in the Netherlands increased resection rates and was associated with better survival after pancreatic-head cancer surgery [23,24]. Higher-volume centres have also achieved textbook outcomes more often, and textbook outcome was associated with improved long-term survival [25]. Volume, therefore, partly represents multidisciplinary selection, pathology quality, recovery, and access to systemic therapy. Centre evaluation should extend beyond technical success to R0 resection, lymph-node yield, 90-day mortality, severe morbidity, FTR, readmission, textbook outcomes, and delivery of planned systemic treatment. Operation-specific evidence on centre volume and rescue is summarised in Table 2.

## 5. Failure to Rescue and the Centre Effect

Complications after pancreatic resection cannot be eliminated, making their recognition and treatment central to the volume–mortality relationship. In a French nationwide analysis of 12,333 pancreatectomies, FTR was 18.3% in low-volume versus 11.9% in high-volume hospitals, and low hospital volume remained independently associated with FTR [26]. A Dutch audit found modest variation in major morbidity but pronounced variation in FTR, with higher adjusted FTR below 30 PDs annually [27]. DP-specific data similarly linked higher pancreatic volume with lower FTR [20]. A 2025 systematic review nevertheless found inconsistent definitions and recommended reporting death after a major postoperative complication [28]. Rescue is a sequence involving surveillance, escalation criteria, experienced radiology, angiography and embolisation, therapeutic endoscopy, critical care, and readiness to reoperate; routine ICU admission alone is insufficient. Among 19,938 pancreatectomies, interhospital transfer after deterioration was associated with substantially higher mortality, with the greatest FTR risk after transfer from a low-volume centre [29]. These findings are consistent with the observation that an experienced surgeon may not offset limited institutional rescue capability and support referral before surgery rather than after physiological deterioration.

## 6. Robotic Pancreatic Surgery

Robotic adoption is particularly relevant to the centre–surgeon volume relationship because it introduces concurrent learning curves at the surgeon, team, and programme levels.

### 6.1. Programme Maturity Before Robotic Adoption

International guidance recommends introducing minimally invasive pancreatic surgery within experienced programmes using structured training, proctoring, stepwise case selection, and outcome monitoring [30,31,32,33]. Robotic DP and RPD have different learning curves, conversion consequences, reconstruction requirements, and rescue needs, making a generic robotic case count inadequate for credentialing. Open PD volume also remains relevant. During a centre’s first five minimally invasive PDs, predicted 90-day mortality was 7.51% with five annual open PDs and 4.39% with 50 [34]. A separate analysis associated at least 20 minimally invasive PDs per year with lower mortality, although 82% of procedures occurred below this threshold [35]. A mature open programme should therefore precede robotic adoption, followed by accumulation of procedure-specific robotic experience.

### 6.2. Surgeon and Centre Learning Curves in Robotic Pancreatoduodenectomy

The learning curve for RPD is prolonged and multidimensional. The Dutch LAELAPS-3 programme used structured training and proctoring, with an initial inflection after approximately 22 RPDs without worsening major morbidity [36]. Follow-up of 635 procedures identified feasibility, proficiency, and mastery phases around 15, 62, and 84 cases [37]. In the international I-MIPS cohort of 2186 RPDs, feasibility was reached after 30–45 cases and proficiency after approximately 90; increasing cumulative experience was associated with improved outcomes, while centres performing more than 20 RPDs annually had lower conversion and reoperation rates and shorter operating time [38].

National evidence further supports distinct but overlapping surgeon- and centre-level learning effects. Mortality was 2.9% in Dutch centres performing at least 20 RPDs annually versus 7.3% below that level [39], while Korean registry data associated more than 20 minimally invasive PDs per year with higher textbook outcomes and lower mortality, conversion, and readmission [40]. In a national electronic health-record study, RPD mortality decreased from 3.9% in a surgeon’s first cases to 0.92% after 19–71 previous procedures, with lower adjusted risk after each doubling of experience [41]. The ten highest-volume US robotic centres performed 41.8% of RPDs and had lower conversion and length of stay, and higher nodal yield than other robotic centres [42]. Together, these findings indicate that cumulative surgeon learning and annual centre repetition are both associated with outcomes during adoption.

### 6.3. Comparative Trials and External Validity

Comparative efficacy cannot be separated from implementation context. LEOPARD-2 was stopped early after complication-related mortality reached 10% with laparoscopic PD versus 2% with open surgery despite minimum centre and surgeon requirements [43]. EUROPA found a similar overall complication burden after robotic and open PD but more grade B/C pancreas-specific complications after RPD [44]. In contrast, the PORTAL phase 3 trial in seven tertiary high-volume centres reported faster recovery and shorter stays with RPD, with similar morbidity but longer operating time and higher expenditure [45]. Participating centres performed more than 100 open PDs and more than 20 RPDs annually, so the findings support RPD after programme maturation rather than during early or low-frequency adoption.

### 6.4. Robotic Distal Pancreatectomy

Robotic DP has a different risk profile and should be assessed separately. DIPLOMA showed non-inferior radical resection for minimally invasive versus open DP for resectable pancreatic cancer, with comparable major outcomes [46], although it did not isolate the robotic approach. In 2403 RDPs at 16 expert centres, progressive learning phases were identified, with improved morbidity and textbook outcomes as experience accumulated and higher annual volume accelerating improvement in operating time [47]. Robotic DP may therefore be more distributable than RPD for standard cases, but still requires procedure-specific experience, conversion readiness, fistula management, and audited outcomes. Evidence on minimally invasive programme volume and learning curves is summarised in Table 3.

## 7. Centralisation, Access, and Time to Treatment

Any potential safety benefit of centralisation depends on patients being able to access the specialised pathway. In the United States, 38.3% of the population lived outside areas with 90-min access to a high-volume pancreatic surgery centre, with disproportionate rural and socially vulnerable exclusion [48]. Access also includes timeliness. From 2006 to 2019, the median diagnosis-to-treatment time for pancreatic cancer increased from 23 to 32 days, and academic or referral hospitals and robotic surgery were associated with longer waits [49]. Travel burden, socioeconomic vulnerability, and waiting time should therefore be audited alongside perioperative outcomes.

Delay must nevertheless be interpreted against care quality. Among 15,310 patients undergoing PD for PDAC, surgery after more than 28 days at a centre performing at least 20 cases annually was associated with lower adjusted five-year mortality than surgery within 14 days at a low-volume centre, together with higher R0 resection rates and lower 30-day mortality [50]. This observational comparison does not imply that delay is beneficial, but suggests that a modest additional wait may be preferable to earlier surgery in a less experienced setting. Networked centralisation can concentrate resection, complex endoscopy, rescue, and robotic governance while retaining diagnosis, prehabilitation, selected systemic therapy, and follow-up closer to home. Hub-and-spoke models and the Italian pancreas-unit framework formalise this approach through minimum activity, multidisciplinary standards, 24-h services, audit, and explicit referral pathways [51,52].

## 8. Proposed Framework for Personalised Referral and Robotic Credentialing

The following framework represents the authors’ interpretation of the reviewed evidence and is intended to support referral and credentialing decisions rather than constitute a clinical guideline or prescriptive standard.

Personalised referral should match the patient and planned operation to the lowest-risk setting with the necessary technical and rescue capabilities rather than justify discretionary decentralisation. Frailty, cardiopulmonary disease, obesity, anticoagulation, pancreatic texture, duct size, vascular involvement, neoadjuvant treatment, and the probability of TP or multivisceral resection increase the value of a mature pancreas centre [22,30,31,32,33]. Standard PD, TP, vascular resection, and RPD during a learning curve should favour an audited high-volume centre with continuous IR, endoscopy, critical care, and reoperation capability [22,26,27,28,29,30,31,32,33,38,39,40]. Selected standard DP may be undertaken more broadly when surgeon experience, fistula pathways, conversion capability, and audited outcomes are adequate, whereas extended, coeliac-axis, or multivisceral DP remains complex pancreatic surgery [19,20,21,46,47].

Within this framework, robotic credentialing should integrate cumulative and recent procedure-specific surgeon exposure; programme maturity, including open pancreatic volume, stable teams, proctoring, and a conversion strategy; audited conversion, grade B/C POPF, PPH, major morbidity, FTR, 90-day mortality, readmission, and oncological quality; and network responsibility for equitable referral, waiting-time surveillance, and rapid transfer [30,31,32,33,34,35,36,37,38,39,40,41,42]. Annual volume can trigger review, but continuation of privileges should depend on risk-adjusted performance and learning-curve status [5,12,30,31,32,33]. Low frequency should prompt proctoring, case-sharing, or referral, while acceptable volume should not shield persistently poor outcomes.

## 9. Limitations and Research Priorities

The evidence is dominated by retrospective administrative and registry studies. High-volume definitions are inconsistent, surgeon and centre volumes are correlated, and residual confounding by referral, tumour biology, socioeconomic status, and case selection is unavoidable [2,4,5,53]. Older studies may overestimate current effects as perioperative systems improve, whereas newer datasets may miss complications treated after transfer. Procedure mixing is an additional limitation because total pancreatectomy volume does not necessarily reflect exposure to PD, RPD, extended DP, or TP, and in-hospital or 30-day mortality misses later pancreatic deaths. Future studies should model operation-specific surgeon and centre volumes simultaneously, use 90-day outcomes, apply standard complication definitions, and incorporate FTR and textbook outcomes. Robotic studies additionally require explicit reporting of the surgeon’s cumulative case number, annual programme volume, prior open experience, training pathway, proctoring, case-selection phase, and conversion policy. Comparative trials should enrol only after predefined proficiency, but should also be complemented by prospective implementation registries that capture the learning phase. Future comparative work should examine pathway completion and cost alongside perioperative outcomes [54].

## 10. Conclusions

Centre and surgeon volume are complementary markers of experience and programme maturity associated with outcomes after pancreatic surgery. Surgeon experience is more closely associated with technical execution, whereas centre-level capability is more consistently associated with complication recognition, rescue, and short-term mortality. These associations appear strongest for PD, TP, and RPD, while standard DP may be less sensitive to volume, although complex DP still requires appropriate technical and rescue expertise. Robotic adoption adds concurrent surgeon, team, and programme learning curves, reinforcing the importance of structured training, programme maturity, and audited outcomes. Referral and credentialing policies should therefore integrate procedure-specific experience, risk-adjusted performance, rescue capability, learning-curve status, and equitable access rather than rely on a single numerical volume threshold.

## Figures and Tables

**Figure 1:**
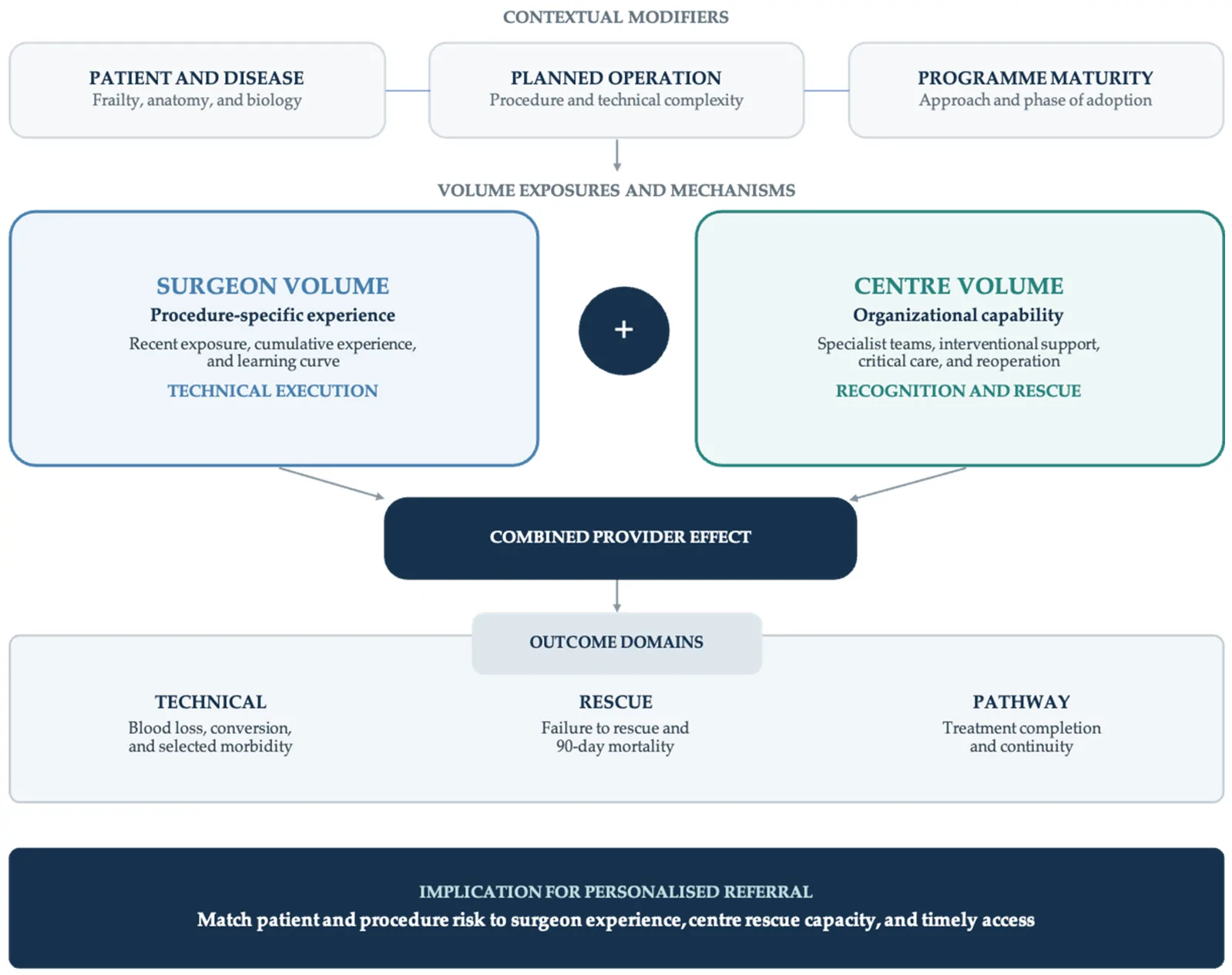
Conceptual framework for interpreting surgeon and centre volume in pancreatic surgery. Surgeon volume primarily reflects procedure-specific experience and technical execution, whereas centre volume primarily reflects organisational capability and rescue infrastructure. Their association with outcomes is modified by patient and disease characteristics, the planned operation, and programme maturity. Personalised referral should integrate these factors with timely access rather than rely on a single annual volume threshold.

**Table 1:** Studies that directly inform centre versus surgeon volume in pancreatic surgery.

Study	Design/Cohort	Volume Exposure	Key Finding	Pancreas-Specific Interpretation
Birkmeyer et al., 2003 [7]	US Medicare; pancreatic resection subgroup	Annual surgeon and hospital volume	Low- versus high-volume surgeon adjusted OR for death 3.61; surgeon volume explained 55% of the hospital-volume association	Both levels matter, but the administrative data predated modern pancreas pathways
Eppsteiner et al., 2009 [15]	Nationwide Inpatient Sample; 3581 pancreatic resections	High-volume surgeon at least 5 resections/year	Adjusted mortality 2.4% versus 6.4%; high surgeon volume associated with lower mortality	Supports an operator-frequency effect after hospital adjustment
Schmidt et al., 2010 [11]	Single high-volume centre; 1003 PDs	Cumulative surgeon experience below versus at least 50; annual volume	Greater cumulative experience associated with lower morbidity, fistula, blood loss, and time; outcomes similar among experienced surgeons	Annual frequency and cumulative technical maturity are not interchangeable
Pecorelli et al., 2012 [16]	Single high-volume centre; 610 PDs	High-volume surgeon at least 12/year	Low-volume surgeons had more fistula, without higher mortality or major morbidity	A mature centre may rescue technical complications
Enomoto et al., 2014 [14]	Nationwide Inpatient Sample; 3137 PDs	Combined hospital and surgeon strata	High surgeon/high hospital volume associated with lower mortality, shorter stay, and lower cost than low/low	Best outcomes when operator and institution are both experienced
Mehta et al., 2016 [13]	Texas Medicare; 2453 pancreatic resections; multilevel models	Surgeon at least 4/year; hospital at least 11/year	High surgeon volume associated with mortality and complications; high hospital volume associated with mortality	Surgeon factors may be more proximal to complication occurrence
Sheetz et al., 2020 [12]	Medicare; 1028 surgeons at 270 hospitals	PD volume and related HPB experience	Only 5.3% performed at least 12 PDs/year; related HPB volume partly compensated for moderate but not very low PD volume	Procedure-specific volume is informative but incomplete for credentialing
Papageorge et al., 2022 [10]	SEER–Medicare; 2450 PDs for PDAC	Four hospital/surgeon strata; propensity matching	Within hospital-volume strata, surgeon volume was not associated with major complications, 90-day mortality, or readmission	Hospital volume was the stronger short-term predictor
Fischer et al., 2023 [5]	Systematic review and meta-analysis	Heterogeneous centre and surgeon cut-offs	High centre volume mortality OR 0.35; high surgeon volume mortality OR 0.29	Both associations are robust, but definitions and adjustment are heterogeneous
Patel et al., 2026 [9]	National Cancer Database; 50,519 PDAC PDs; 3155 surgeons; 976 hospitals	Hospital at least 20/year; surgeon at least 12/year	90-day mortality 6.4% low/low, 5.7% high surgeon/low centre, 4.0% low surgeon/high centre, and 3.5% high/high	A high-volume surgeon did not compensate for a low-volume centre

Note: Thresholds are study-specific and should not be interpreted as universal credentialing standards. HPB, hepato-pancreato-biliary; OR, odds ratio; PD, pancreatoduodenectomy; PDAC, pancreatic ductal adenocarcinoma.

**Table 2:** Operation-specific centre-volume and rescue evidence.

Operation	Study	Design/Cohort	Key Finding	Implication
PD	Hata et al., 2016 [3]	Meta-analysis; 58,023 PDs	Higher hospital volume associated with lower postoperative mortality	Strongest procedure-specific evidence for centralisation
PD	van der Geest et al., 2016 [18]	Netherlands; 3420 PDs for cancer	90-day mortality decreased across centre-volume strata; highest-volume centres had better nodal assessment and adjuvant treatment	Centre volume captures the full oncological pathway
PD	Liu et al., 2018 [17]	England; 14,935 PDs	No mortality difference between 36–60 and more than 60 PDs/year after substantial temporal improvement	Possible plateau with residual case-mix differences
All pancreatectomy	El Amrani et al., 2018 [26]	France; 12,333 pancreatectomies	FTR 18.3% low versus 11.9% high volume; low volume independently associated with FTR	Rescue is a major mechanism of the centre effect
PD	van Rijssen et al., 2018 [27]	Dutch audit; nationwide PD	Mortality variation reflected FTR more than major-complication incidence; fewer than 30 PDs/year associated with higher FTR	Complication recognition and treatment distinguish centres
DP	Roussel et al., 2019 [19]	France; 3314 DPs	No volume cut-off and no significant mortality difference across volume groups	Standard DP may be less mortality-sensitive to volume
DP	Lequeu et al., 2021 [20]	France; 10,632 DPs	At least 20 pancreatectomies/year associated with lower mortality and FTR	Rescue may reveal a centre effect not seen with mortality alone
TP	Latenstein et al., 2022 [22]	Prospective European study; 277 TPs at 43 centres	Mortality 4% at centres with at least 60 PDs/year versus 10% below; lower PD volume independently associated with death	TP should be concentrated in mature PD programmes
PD and DP	Vawter et al., 2023 [21]	ACS-NSQIP; 30,357 PDs and 14,800 DPs	Pancreas-targeted hospitals had lower PD mortality, morbidity, and FTR; differences did not extend to DP	Volume policy should be operation-specific
PDAC resections	Kalagara et al., 2022 [25]	National Cancer Database; 7270 resections	Textbook outcome 23.4% low-volume versus 37.5% high-volume; textbook outcome associated with survival	Quality measures can mediate the volume–survival association

Note: ACS-NSQIP, American College of Surgeons National Surgical Quality Improvement Program; DP, distal pancreatectomy; FTR, failure to rescue; PD, pancreatoduodenectomy; PDAC, pancreatic ductal adenocarcinoma; TP, total pancreatectomy.

**Table 3:** Robotic and minimally invasive pancreatic surgery—programme volume and learning curves.

Domain	Study	Design/Setting	Key Finding	Interpretation
MIPD adoption	Torphy et al., 2021 [34]	NCDB implementation analysis	Higher open PD annual volume protected during early MIPD; predicted mortality in first five cases 7.51% at 5 versus 4.39% at 50 open PDs/year	A mature open programme should precede robotic PD
MIPD volume	Conroy et al., 2022 [35]	3079 MIPDs	At least 20 MIPDs/year associated with lower mortality; 82% performed below the threshold	Low-frequency diffusion remains a safety concern
Laparoscopic PD	van Hilst et al., 2019 [43]	LEOPARD-2 randomised trial	Stopped early; complication-related mortality 10% laparoscopic versus 2% open despite training and minimum volume criteria	Minimum credentials alone do not ensure safe implementation
RPD training	Zwart et al., 2022 [36]	LAELAPS-3; 275 RPDs, 15 surgeons, 7 centres	Structured training and proctoring; initial inflection near 22 RPDs without worsening major morbidity	Proctored team-based implementation can control early risk
RPD learning	Zwart et al., 2023 [37]	635 RPDs after multicentre training	Feasibility, proficiency, and mastery phases around 15, 62, and 84 procedures	Competence is progressive, not binary
RPD learning	Jones et al., 2024 [38]	I-MIPS; 2186 RPDs at 18 centres	Feasibility 30–45 and proficiency about 90; more than 20 RPDs/year associated with lower conversion and reoperation	Cumulative surgeon learning and annual centre repetition both matter
RPD volume	de Graaf et al., 2024 [39]	Dutch nationwide matched analysis	Centres with at least 20 RPDs/year had mortality 2.9% versus 7.3% and conversion 6.3% versus 11.2%	Nationwide implementation remained centre-volume sensitive
MIPD volume	Kwon et al., 2024 [40]	1552 MIPDs at 10 Korean centres	More than 20/year associated with higher textbook outcome and lower mortality, conversion, and readmission	Centre frequency influences composite and rescue outcomes
RPD surgeon learning	Donnelly et al., 2026 [41]	National EHR; 23,995 PDs including 1578 RPDs	RPD mortality declined from 3.9% in first cases to 0.92% after 19–71 prior cases; risk fell with each doubling of experience	Early national adoption carries a measurable surgeon learning effect
RPD efficacy	Klotz et al., 2024 [44]	EUROPA exploratory randomised trial	Overall complication burden similar; more grade B/C pancreas-specific complications after RPD	Technical advantages do not eliminate pancreatic morbidity
RPD efficacy	Jin et al., 2026 [45]	PORTAL phase 3 RCT; seven tertiary high-volume centres	Faster recovery and shorter stay with similar morbidity; longer time and higher cost	Benefits were demonstrated after credentialing in mature centres
RDP learning	Müller et al., 2026 [47]	2403 RDPs at 16 expert centres	Competency, proficiency, and mastery around 46, 63, and 73 procedures	Distal robotic resection also has a programme learning curve
MIDP efficacy	Korrel et al., 2023 [46]	DIPLOMA randomised trial	Minimally invasive DP was non-inferior for radical resection with comparable major outcomes	Supports trained MIDP but does not remove operation-specific governance

Comparative efficacy and implementation safety are separated. Results from credentialled high-volume programmes should not be extrapolated directly to early low-frequency adoption. EHR, electronic health record; I-MIPS, International Consortium on Minimally Invasive Pancreatic Surgery; MIDP, minimally invasive distal pancreatectomy; MIPD, minimally invasive pancreatoduodenectomy; NCDB, National Cancer Database; PD, pancreatoduodenectomy; RCT, randomised controlled trial; RDP, robotic distal pancreatectomy; RPD, robotic pancreatoduodenectomy.

## Data Availability

No new data were created or analysed in this study. Data sharing is not applicable to this article.

## Supplementary Materials

The following supporting information can be downloaded at https://www.mdpi.com/article/10.3390/jpm16100502/s1: Supplementary Methods S1. Reproducible PubMed/MEDLINE search strategies for the narrative review.

## Abbreviations

DP, distal pancreatectomy; FTR, failure to rescue; HPB, hepato-pancreato-biliary; ICU, intensive care unit; IR, interventional radiology; MIDP, minimally invasive distal pancreatectomy; MIPD, minimally invasive pancreatoduodenectomy; PD, pancreatoduodenectomy; PDAC, pancreatic ductal adenocarcinoma; POPF, postoperative pancreatic fistula; PPH, postpancreatectomy haemorrhage; RDP, robotic distal pancreatectomy; RPD, robotic pancreatoduodenectomy; TP, total pancreatectomy.
