# Supplementary material for: Centre Versus Surgeon Volume in Pancreatic Surgery
Source: J Pers Med. 2026 Sep 28;16(10):502. doi: 10.3390/jpm16100502 (PMC13705692; doi:10.3390/jpm16100502)
Supplement: Supplementary file 1 [file jpm-16-00502-s001.zip › jpm-4564509-supplementary.pdf]

Supplementary Methods S1. PubMed/MEDLINE search strategy

The literature search was conducted in PubMed/MEDLINE from database inception through 24 August 2026. As this study was designed as a critical narrative review rather than a systematic review, searching was iterative and concept-based rather than based on a single prospectively locked Boolean strategy. The following reproducible searches reflect the principal concept blocks used to identify evidence concerning centre and surgeon volume, centralisation and failure to rescue, robotic and minimally invasive programme maturity, and access to pancreatic surgical care.

Search 1. Centre and surgeon volume in pancreatic surgery

```
(
  pancreatectom*[tiab]
  OR "pancreatic resection"[tiab]
  OR "pancreatic resections"[tiab]
  OR pancreatoduodenectom*[tiab]
  OR pancreaticoduodenectom*[tiab]
  OR Whipple[tiab]
  OR "distal pancreatectomy"[tiab]
  OR "distal pancreatectomies"[tiab]
  OR "total pancreatectomy"[tiab]
  OR "total pancreatectomies"[tiab]
)
AND
(
  "hospital volume"[tiab]
  OR "centre volume"[tiab]
  OR "center volume"[tiab]
  OR "surgeon volume"[tiab]
  OR "operator volume"[tiab]
  OR "provider volume"[tiab]
  OR "case volume"[tiab]
  OR "procedure volume"[tiab]
  OR "annual volume"[tiab]
  OR "high-volume"[tiab]
  OR "low-volume"[tiab]
  OR centralisation[tiab]
  OR centralization[tiab]
  OR regionalisation[tiab]
  OR regionalization[tiab]
)
AND ("1800/01/01"[dp] : "2026/08/24"[dp])
```

Search 2. Failure to rescue and organisational capability

```
(
  pancreatectom*[tiab]
  OR "pancreatic resection"[tiab]
  OR "pancreatic resections"[tiab]
  OR pancreatoduodenectom*[tiab]
  OR pancreaticoduodenectom*[tiab]
  OR "distal pancreatectomy"[tiab]
  OR "total pancreatectomy"[tiab]
)
AND
(
  "failure to rescue"[tiab]
```

```
  OR "failure-to-rescue"[tiab]
  OR "textbook outcome"[tiab]
  OR "interhospital transfer"[tiab]
  OR "inter-hospital transfer"[tiab]
  OR "hospital transfer"[tiab]
  OR rescue[tiab]
)
AND
(
  volume[tiab]
  OR centralisation[tiab]
  OR centralization[tiab]
  OR regionalisation[tiab]
  OR regionalization[tiab]
  OR hospital[tiab]
  OR centre[tiab]
  OR center[tiab]
)
AND ("1800/01/01"[dp] : "2026/08/24"[dp])
```

Search 3. Minimally invasive and robotic pancreatic surgery, learning curves, and programme maturity

```
(
  pancreatectom*[tiab]
  OR "pancreatic resection"[tiab]
  OR pancreatoduodenectom*[tiab]
  OR pancreaticoduodenectom*[tiab]
  OR "distal pancreatectomy"[tiab]
)
AND
(
  robotic[tiab]
  OR "robot-assisted"[tiab]
  OR "robot assisted"[tiab]
  OR "minimally invasive"[tiab]
  OR laparoscopic[tiab]
)
AND
(
  "learning curve"[tiab]
  OR "learning curves"[tiab]
  OR proficiency[tiab]
  OR competency[tiab]
  OR mastery[tiab]
  OR training[tiab]
  OR proctor*[tiab]
  OR credential*[tiab]
  OR implementation[tiab]
  OR adoption[tiab]
  OR "case volume"[tiab]
  OR "annual volume"[tiab]
  OR "centre volume"[tiab]
  OR "center volume"[tiab]
  OR "programme volume"[tiab]
  OR "program volume"[tiab]
)
AND ("1800/01/01"[dp] : "2026/08/24"[dp])
```

Search 4. Centralisation, referral, access, and time to treatment

```
(
  "pancreatic surgery"[tiab]
  OR "pancreatic cancer"[tiab]
  OR pancreatectom*[tiab]
  OR "pancreatic resection"[tiab]
  OR pancreatoduodenectom*[tiab]
  OR pancreaticoduodenectom*[tiab]
)
AND
(
  centralisation[tiab]
  OR centralization[tiab]
  OR regionalisation[tiab]
  OR regionalization[tiab]
  OR referral[tiab]
  OR access[tiab]
  OR accessibility[tiab]
  OR travel[tiab]
  OR distance[tiab]
  OR "waiting time"[tiab]
  OR "waiting times"[tiab]
  OR "time to treatment"[tiab]
  OR "hub-and-spoke"[tiab]
  OR "hub and spoke"[tiab]
  OR network*[tiab]
)
AND ("1800/01/01"[dp] : "2026/08/24"[dp])
```

Reference lists of relevant systematic reviews, international guidelines, consensus statements, and key comparative studies were additionally screened for eligible evidence, followed by forward citation searching. Evidence selection and prioritisation followed the criteria described in the main manuscript.
